# Sleep and Multisystem Biological Risk: A Population-Based Study

**DOI:** 10.1371/journal.pone.0118467

**Published:** 2015-02-25

**Authors:** Judith E. Carroll, Michael R. Irwin, Sharon Stein Merkin, Teresa E. Seeman

**Affiliations:** 1 Cousins Center for Psychoneuroimmunology, Semel Institute of Neuroscience and Human Behavior, David Geffen School of Medicine, University of California Los Angeles, Los Angeles, California, United States of America; 2 Division of Geriatrics, David Geffen School of Medicine, University of California Los Angeles, Los Angeles, California, United States of America; University of Alabama at Birmingham, UNITED STATES

## Abstract

**Background:**

Short sleep and poor sleep quality are associated with risk of cardiovascular disease, diabetes, cancer, and mortality. This study examines the contribution of sleep duration and sleep quality on a multisystem biological risk index that is known to be associated with morbidity and mortality.

**Methods:**

Analyses include a population-based sample from the Midlife Development in the United States survey recruited to the Biomarker substudy. A total of 1,023 participants aged 54.5 years (SD = 11.8), 56% female and 77.6% white, were included in the analyses. A multisystem biological risk index was derived from 22 biomarkers capturing cardiovascular, immune, lipid-metabolic, glucose-metabolic, sympathetic, parasympathetic, and hypothalamic-pituitary-adrenal systems. Self-reported average sleep duration was categorized as short (<5 hrs), below normal (5 to <6.5 hrs), normal (6.5 to <8.5 hrs), and long sleepers (8.5+ hrs). Sleep quality was determined using the Pittsburgh Sleep Quality Index categorized as normal (≤5) and poor quality (>5) sleep.

**Findings:**

Linear mixed effect models adjusting for age, gender, race, education, income, BMI, and health status were performed. As compared to normal sleepers, multisystem biological risk in both short (B(SE) = .38(.15), *p*<.01) and long sleepers (B(SE) = .28(.11), *p*<.01) were elevated. Poor quality sleep alone was associated with elevated multisystem biological risk (B(SE) = .15(.06), *p* = .01), but was not significant after adjustment for health status. All short sleepers reported poor sleep quality. However in the long sleepers, only those who reported poor sleep quality exhibited elevated multisystem biological risk (B(SE) = .93(.3), *p* = .002).

**Conclusions:**

Self-reported poor sleep quality with either short or long sleep duration is associated with dysregulation in physiological set points across regulatory systems, leading to elevated multisystem biological risk. Physicians should inquire about sleep health in the assessment of lifestyle factors related to disease risk, with evidence that healthy sleep is associated with lower multisystem biological risk.

## Introduction

Insufficient sleep is a major public health epidemic [1,2], with estimates that between 9 and 13% of adults in the United States are not getting enough sleep [3,4]. The prevalence of inadequate sleep in employed adults is higher, with approximately 30% reporting short sleep duration (CDC) [1]. This is a particular concern given the growing evidence that short sleep duration increases risk for disease and death [5–7]. By contrast, excessive sleeping has also been related to increased risk, with speculation that long sleep may be related to risk primarily because it serves as a proxy indicator that an individual has sleep fragmentation and/or failing health [5,6,8]. In a recent meta-analysis, both short and long sleep duration are significant predictors of all-cause mortality, including cardiovascular and non-cardiovascular deaths.^5^ However, a majority of these findings [sleep duration-mortality relationship] do not consider other dimension of sleep that contribute to sleep quality. Poor sleep quality (e.g. difficulty falling asleep, staying asleep, early awakening) has itself been related to risk. However, this relationship is particularly pronounced when occurring with short sleep duration, in which the combination of poor sleep quality and short sleep duration predicts higher risk for hypertension, type 2 diabetes, and mortality than either poor sleep quality or short sleep duration alone [9–12]. Likewise, poor sleep quality among long sleepers is predictive of cardiovascular disease [12,13]. Additional research is needed to characterize the role of sleep quality in both short and long sleepers in relation to biomarkers of disease risk.

The mechanisms through which short and long sleep duration, particularly when combined with poor sleep quality, contribute to elevated risk are not clear, but are likely multidimensional. In the case of short sleep duration and poor quality sleep, a prevailing supposition is that insufficient sleep, either of reduced absolute length or of poor quality, acts as a physiological stressor, impacting neurobiological regulation of circadian rhythm [14]. This physiological stress leads to a greater burden on the regulatory systems to maintain allostasis under adverse conditions, which causes wear and tear across systems, termed *allostatic load* [14–16]. Allostatic load is witnessed by shifts in regulatory set points that imply cumulative burden on those systems. Some examples of shifts in set points include elevated resting blood pressure, increased glucose, increased autonomic imbalances, alterations in diurnal patterns of cortisol, and inflammation [15,17,18]. Under experimentally induced short sleep duration, either acute sleep deprivation or sleep restriction for several days, are reported to cause temporary changes in biomarkers for the metabolic, endocrine, autonomic, cardiovascular, and immune systems [19–31]. Similarly, sleep fragmentation, a major component of poor sleep quality, has also been shown to impact many of these systems [32–35]. Chronic short sleep duration, particularly when combined with poor sleep quality [36], may have cumulative effects with a large body of research documenting associations of short sleep with higher rates of metabolic syndrome [7], obesity [37], diabetes [38,39], dyslipidemia, increased proinflammatory profiles [40,41], and elevated blood pressure [42–44]. Chronic poor sleep quality has also been linked to many of these health outcomes, including diabetes [45,46], inflammation [47,48], metabolic syndrome [49], and cardiovascular disease and death [12,13,50]. Short sleep duration is thought to put physical stress on neuroendocrine and automatic regulatory systems [14], with evidence of short sleep being related to shifts in diurnal cortisol rhythms and altered sympathovagal balance, with elevated sympathetic and reduced parasympathetic control [38,51–53]. Sleep fragmentation is also thought to modify these neuroendocrine and autonomic regulatory systems [32–35]. Moreover, Vgontzas and colleagues work argues that short sleep when combined with poor sleep quality (i.e., insomnia complaints) is particularly detrimental to these systems and elevates disease risk [54–57]. Taken together this work suggests that not getting adequate and good quality sleep impacts regulatory dynamics across neuroendocrine, sympathovagal, cardiovascular, metabolic, and immune systems, and might contribute to multisystem biological risk.

The majority of research to date has focused on each system separately, despite evidence that these multiple physiological systems interact with each other [11] and alter the cumulative whole. A multisystem approach is grounded in evidence that the multiple routes are likely to contribute to disease, which together acts as a powerful epidemiological predictor of morbidity and mortality outcomes [16,17,58–61]. To date, two studies have evaluated sleep in relations to multisystem risk using eight to nine biomarker to capture allostatic load, and report that short sleep [62,63], particularly with poor sleep quality [63], and insomnia symptoms [62] related to elevated allostatic load. These studies included biomarkers of the cardiovascular, metabolic, and immune systems, but did not assess sympathovagal or neuroendocrine parameters. The present analysis are the first to consider the cumulative effects across seven biological regulatory systems, and similar to these previous reports, we approach the analyses using a multisystem perspective.

Consistent with existing evidence linking short sleep duration with elevated risk within individual systems, we hypothesized that individuals with short sleep duration would have greater multisystem biological risk scores, indicative of higher allostatic load independent of chronic health conditions. In addition, we examine the association between long sleep and multisystem biological risk. Whereas long sleep is associated with increased health risks, it has been suggested that this relationship reflects underlying problems in health and/or sleep quality that increases demands for sleep for a longer period of time, as opposed to long sleep alone leading to health declines [5,6,8]. Hence, we further hypothesize that the associations between long sleep and elevated multisystem biological risk scores would be present in the long sleepers with poor sleep quality but not with normal sleep quality. In addition, since sleep quality and depression have both independent and overlapping effects on risk [64], we examine the effects after further adjustment for depression. We test these hypotheses in a large community sample of midlife adults from the United States of America.

## Methods

### Participants

As was previously reported [65–68], the Midlife Development in the United States (MIDUS) survey was conducted in 1995 to 1996 and consisted of a random-digit telephone dialing of potential respondents aged 25–74 years across the 48 contiguous states, including an over-sampling of twin pairs and siblings. In 2004 to 2006, a follow up was completed (MIDUS II), with 75% of MIDUS I participating. The present set of analyses includes a subset of MIDUS II participants who agreed to be in the Biomarkers substudy [68]. A total of 1255 participants came for an overnight stay at a general clinical research center (GCRC) housed at the University of California, Los Angeles, Georgetown University, and the University of Wisconsin, Madison. Institutional review board monitoring and approval was obtained from the University of California Los Angeles Institutional Review Board, Georgetown University Institutional Review Board, and the University of Wisconsin Health Sciences Institutional Review Board. All participants provided written informed consent. A detailed comparison of participants in the Biomarker substudy with the main sample respondents can be found elsewhere [68]. The present analyses include 1,023 participants who took part in the Biomarker substudy, reported no regular use of medication for sleep, provided biomarkers to compute multisystem biological risk and completed questionnaire on sleep, depression, self-evaluated health, and other psychosocial, behavioral, and health questionnaires.

### Procedures

Demographic information was obtained during an initial telephone interview. After arrival at the GCRC, participants completed a medical history, health and behavior questionnaires, and underwent a physical examination. Fasting morning blood was collected after a night of sleep for the measurement of blood derived biomarkers. A 12-hour overnight protocol for the collection of urine was followed from 7:00 PM to 7:00 AM. Resting heart-rate variability was obtained the morning following breakfast. All collection was done following standardized protocols across sites [68].

### Measures

**Sleep duration.** Average night time sleep duration over the prior month was reported by subjects using a standard question from the Pittsburgh Sleep Quality Index (PSQI) [69]. Consistent with the epidemiological literature examining sleep duration with morbidity and mortality [5,6], we categorized sleep duration into four groups: Less than 5 hours, 5 to less than 6.5 hours, 6.5 to less than 8.5 hours, and 8.5 hours or more.

*Sleep Quality*. The Pittsburgh Sleep Quality Index [69] was used to derive a global score of sleep quality across seven domains. Scores greater than 5 indicate poor sleep quality and elevated sleep disturbances [69]. We categorized scores as follows: 5 or less = normal sleep, greater than 5 = poor sleep. Regular use of sleep medication was determined by self-report of using medications 3 or more times per week reported either on the PSQI or during medical interview.

**Multisystem Biological Risk Index (Allostatic Load).** A total of 22 biomarkers were measured and included in the allostatic load score, representative of 7 different physiological systems. This included the cardiovascular system (systolic blood pressure, pulse pressure, and heart rate), lipid metabolic system (triglycerides, high density lipoprotein (HDL), low density lipoprotein (LDL), body mass index, and waist-hip ratio), glucose metabolic system (fasting blood glucose, glycosylated hemoglobin, and the homeostasis model of assessment of insulin resistance (HOMA-IR)), immune system (inflammatory markers: C-reactive protein, Interleukin(IL)-6, e-Selectin, intracellular adhesion molecule-1, and fibrinogen), sympathetic nervous system (SNS; Urinary norepinephrine and epinephrine), parasympathetic nervous system (PNS; heart rate variability: standard deviation of R-R intervals, low frequency (LF) and high frequency (HF) spectral power), and hypothalamic-pituitary-adrenal system (urinary cortisol and serum dehydroepiandrosterone sulfate (DHEA-S)). The measurement methods have been reported in detail in prior publications [65,67,68]. Multisystem biological risk was computed by calculating a 0–1 risk score within each system, which reflects the proportion of biomarkers within that system for which the participant’s values fall in the highest-risk quartile. The seven proportional scores were then summed, with final risk scores ranging from 0–7. This summation allows for equal weighting of each system in the multisystem biological risk computation regardless of the number of biomarkers assessed within a system. Cut points for individual biomarkers can be found in a previous report [67]. In addition, use of medication prescribed to lower specific biomarkers of risk was considered an indication of dysregulation within that system (e.g. anti-hypertensive medication indicates increased risk in the cardiovascular system). We scored individuals as being in the high risk quartile of that biomarker for which the medication is known to target. This included medications for lowering blood pressure, heart rate, glucose, cholesterol, and triglycerides. We also performed sensitivity analyses using a risk score that did not include medication in the computation. The individual system score was computed if participants had half or more of the biomarkers for that system. A total of 116 participants had only one missing system scores (91 missing PNS scores; 25 missing one of the other system scores). Data were missing at random for all the systems except parasympathetic. For the parasympathetic, missingness was more common in older adults and was due to instrumentation problems that led to poor quality data signals. For 91 patients who were missing only the parasympathetic system score, we used single mean conditional imputation that replaces missing values with predicted values from a regression equation generated with non-missing data [70]. This equation estimates missing risk scores using participants’ scores on the other six systems, age, gender, and race/ethnicity as predictors. The others missing system scores values were imputed with the modal value, which was zero. Individuals had to have 6 of the 7 system scores to have a risk score computed. Bivariate associations of subscale scores of the multisystem biological risk index are reported in S1 Table.

**Additional Variables.** In addition to the basic demographic factors (age, gender, race), our analyses adjust for indicators of socioeconomic status, including education obtained (≤ high school, some college, vs. ≥ college) and income/poverty ratio, and BMI. Chronic condition scores of 0–8 indicate the number of reported conditions that might influence sleep (including lung condition, cardiovascular disease, history of stroke, history of cancer, joint or bone ailments, gastrointestinal distress, thyroid diseases, and/or neurological disorder). Vitality was assessed by a three item scale asking participants to rate using a likert scale from 1 to 5: their energy levels compared to five years ago, and rate how much they feel full of life and active. Scale score ranges from 3 to 15 with higher scores indicating higher vitality. Self-evaluated physical health was assessed with a single question asking participants to rate their physical health as either poor = 1, fair = 2, good = 3, very good = 4, excellent = 5. As clinical depression is associated with an increase in disease risk [71], and may contribute to the sleep and disease relationship [64], the Center for Epidemiologic Studies Depression (CESD) scale [72] scores of 16 or greater were used to identify probable clinical depression based on established cutoffs with high clinical sensitivity and specificity [73,74].

### Statistical Analyses

Statistical analyses were performed using IBM SPSS statistics (v.21). Initial analyses tested for differences in means or percentage within category in demographics and chronic conditions across the four sleep duration categories, which was performed using ANOVA followed by pairwise comparisons or chi squared test. Linear associations of covariates with multisystem biological risk scores were determined with Pearson correlation. Tests for differences in multisystem biological risk between sleep duration categories and sleep quality categories were performed using a linear mixed effects model that included a random intercept at the family level (MIDUS includes twins and siblings) to account for within family correlations between siblings and twins in the study sample (sibling/twin pair (n = 107) ICC = .50 indicated a relatively high within group similarity). Model 1 adjusts for age, gender, race (white vs. non-white), education, income to poverty ratio, and BMI (to adjust for any residual variance accounted for by BMI), and tests for differences between: 1) sleep categories using normal duration (6.5 to <8.5 hours per night) as the reference, and 2) sleep quality groups using normal quality sleepers as the reference. In addition to adjusting for Model 1 covariates, Model 2 adjusts for comorbid chronic conditions and self-evaluated physical health. Secondary mixed linear effect models using case selection explore differences in long sleepers with or without poor sleep quality and further adjusting for depression, and examine differences by sleep duration category in the good sleep quality group.

## Results

Descriptive statistics of the sample, along with statistics by sleep category, can be found in Table 1. Differences between sleep categories in demographic factors were present, with a greater percentage of Whites sleeping longer than non-Whites. Average BMI differed by sleep category with short sleepers having the highest mean BMI. Poor sleep quality was more likely to occur in non-Whites and women (*p*’s < .05). Poor sleepers compared to normal sleepers had significantly lower average age, higher BMI, more chronic conditions, poorer self-rated health, lower vitality, higher depressive symptoms, and lower income to poverty ratio (all *p*’s < .05).

Correlation analyses showed that higher multisystem biological risk was significantly associated with older age (r = .44, *p* < .001), higher BMI (r = .34, *p* < .001), more chronic conditions (r = .25, *p* < .001), poorer vitality (r = -.13, *p* < .001), a trend for higher depressive symptoms scores (r = .06, *p* < .08), and poorer self-evaluated health (r = -.30, *p* < .001). Lower educational attainment was associated with significantly higher multisystem biological risk (*p* < .001).

**Table 1:** Means (Standard Deviations) or percentages by sleep duration category.

	Total Sample	< 5 hrs.	5 to < 6.5 hrs.	6.5 to < 8.5 hrs.	≥ 8.5	P value
	n = 1023	n = 42	n = 260	n = 635	n = 86	
Age	54.5(11.8)	52.4(10.7)	52.7(11.5)	55.0(11.8)	56.7(12.2)	0.007^a^
Race						<0.0001
% of White	77.6%	52.4%	74.2%	80%	86%	
% of Non-White	22.4%	47.6%	25.8%	20%	14%	
Gender						0.75
% Male	44%	42.9%	43.1%	45.2%	39.5%	
% Female	56%	57.1%	56.9%	54.8%	60.5%	
Body Mass Index (BMI)	29.7(6.6)	31.4(8.6)	30.5(7.2)	29.4(6.3)	28.7(5.4)	0.02 ^b^
Income Poverty Ratio	5.2(4.2)	4.1(3.7)	5.1(4.1)	5.4(4.2)	5.0(4.2)	0.21
Education						0.20
≤ High School	27.4%	31%	30.8%	25.2%	31.4%	
Some College	29.9%	35.7%	31.9%	28.8%	29.1%	
≥ College Degree	42.7%	33.3%	37.3%	46%	39.5%	
Chronic Conditions (0–8)	1.1(1.1)	1.4(1.2)	1.1(1.1)	1.1(1.1)	1.2(1.1)	0.25
Self-Rated Health (1–5, 1 = Poor)	3.7(.95)	3.4(1.1)	3.5(1.0)	3.8(.9)	3.5(1.0)	<0.001^c^
CESD Depression (% 16 or greater)	13.7%	33.3%	20.4%	9.6%	14%	<0.001
PSQI Global (% >5)	40.4%	100%	69.9%	27.7%	15.4%	<0.001

^a^ Difference at *p* < .05: 6.5 to <8.5 vs. 5 to <6.5, ≥ 8.5 vs. 5 to <6.5.

^b^ Difference at *p* < .05: <5 to ≥ 8.5, 5 to <6.5 vs. 6.5 to < 8.5, ≥ 8.5 vs. 5 to <6.5

^c^ Difference at *p* <.05: 6.5 to <8.5 vs. all other categories.

### Sleep Duration and Multisystem Risk

**Fig 1:**
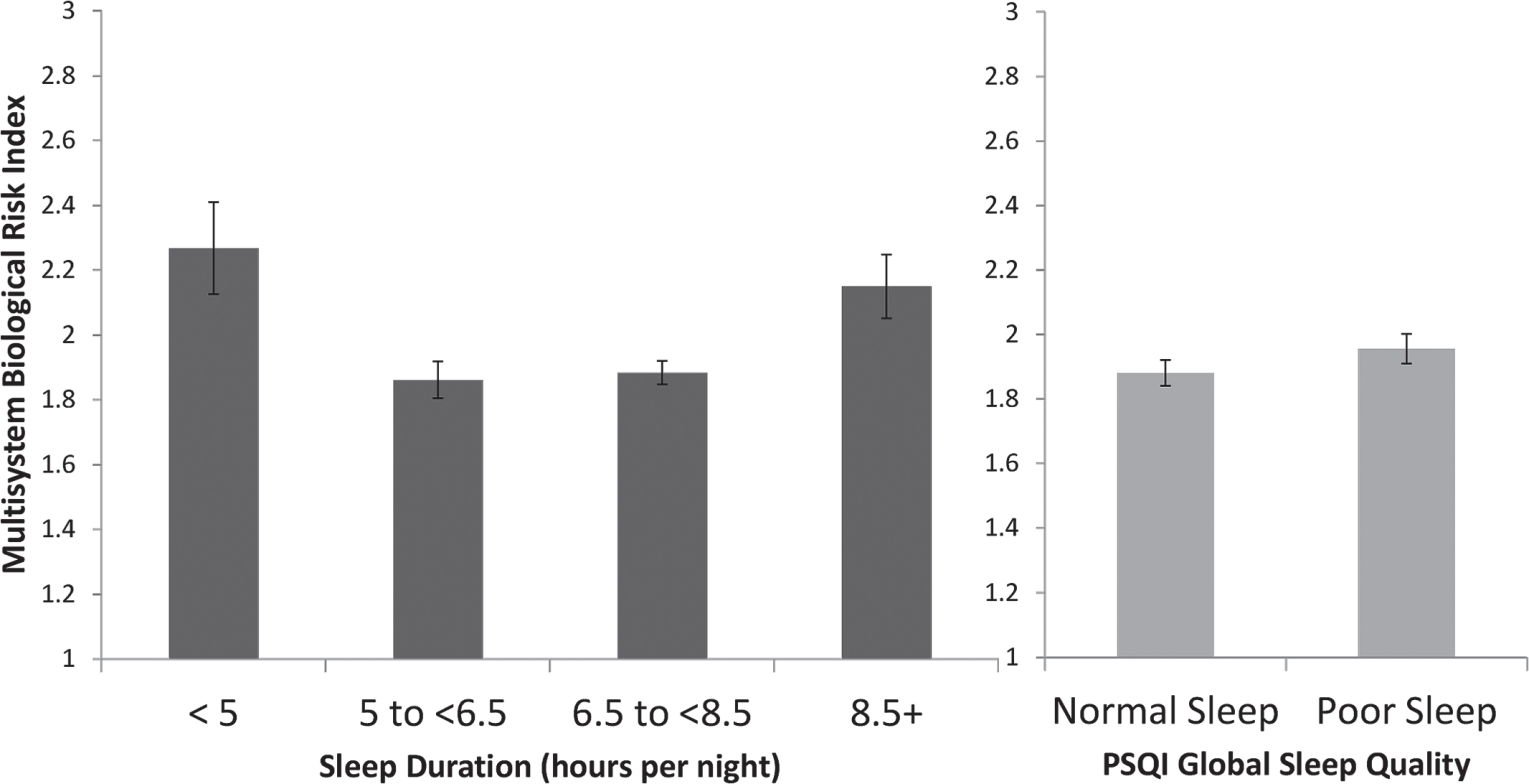
Estimated mean and standard error of multisystem biological risk by sleep duration (1a) and PSQI global sleep score (1b). Mean and standard error estimates derived from model after adjustments by age, gender, race, BMI, education, income poverty ratio, chronic conditions, and self-evaluated physical health. Multisystem Biological Risk score ranged from 0–7.

As compared to normal sleepers, linear mixed effect analyses adjusting for age, gender, race, education, income to poverty ratio, and BMI revealed that multisystem biological risk scores were elevated in both short (B(SE) = .43(.15), *p* = .004) and long sleepers (B(SE) = .34(.11), *p* = .001). Means by group are displayed in Fig. 1. Tests for the interaction of age with sleep duration and gender with sleep duration was not significant.

Next we ran a separate model testing for the effect of sleep duration on multisystem biological risk after adjustment by health status (Model 2: chronic conditions and self-reported physical health; See Table 2). After adjusting for health status, the difference in multisystem biological risk in both short sleepers and long sleepers compared to normal sleepers remained significant, *p* < .01. We ran sensitively analyses, excluding 31 participants with HIV and neurological conditions and found no difference in the findings.

**Table 2:** Mixed linear effect model coefficients (B) for multisystem biological risk by sleep quality and duration.

	PSQI Normal vs. Poor Quality Sleep		Short vs. Normal Sleep Duration		Long vs. Normal Sleep Duration	
	B(SE)	*p* value	B(SE)	*p* value	B(SE)	*p* value
**Unadjusted**	.17(.07)	.02	.47(.18)	.009	.38(.13)	.003
**Model 1**	.15(.06)	.01	.43(.15)	.004	.34(.11)	.001
**Model 2 Health status**	.09(.06)	.16	.38(.15)	.009	.28(.11)	.008

*Model 1*: *age, gender, race, BMI, education, income poverty ratio*

*Model 2*: *age, gender, race, BMI, education, income poverty ratio, chronic conditions, self-evaluated health*

### Sleep Quality and Multisystem Biological Risk

As compared to those with normal sleep quality, linear mixed effect analyses adjusting for age, gender, race, education, income to poverty ratio, and BMI revealed that participants with poor sleep quality had elevated multisystem biological risk scores, B(SE) = .15(.06), *p* = .01. Results were no longer significant after adjustment for health status, B(SE) = .09 (.06), *p* = .16.

### Poor Quality Sleep and Sleep Duration

All short sleepers reported poor sleep quality, and there were no cases of short sleep and good quality sleep. Due to concerns that short sleep duration might contribute to higher PSQI scores, we also computed the PSQI without the sleep duration item. Among the 42 subjects in the group who were short sleepers with poor sleep quality, only 4 subjects were re-classified when the sleep duration item was no longer considered in determining poor sleep quality. Hence, in the short sleepers who have high PSQI scores, poor sleep quality is not driven by the single item related to sleep duration.

**Fig 2:**
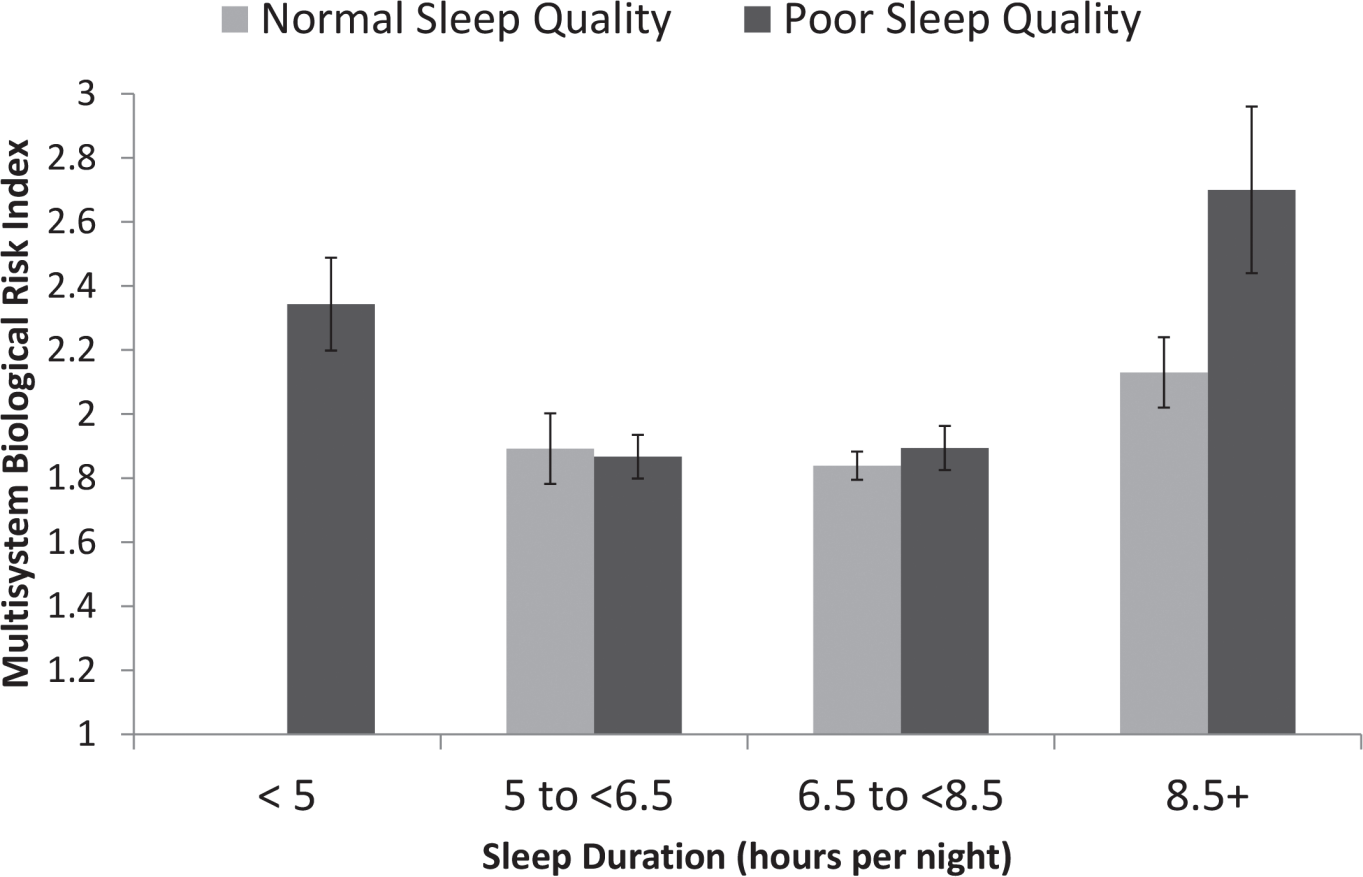
Estimated mean and standard error of multisystem biological risk by sleep duration and quality. Mean and standard error estimates derived from model after adjustments by age, gender, race, BMI, education, income poverty ratio, chronic conditions, and self-evaluated physical health. Multisystem Biological Risk score ranged from 0–7.

To examine whether the elevated multisystem biological risk differed in long sleepers who reported either normal or poor sleep quality, we stratified the long sleep duration group (n = 86) on the basis of sleep quality scores. As compared to long sleepers with normal sleep quality, long sleepers reporting poor sleep quality had significantly elevated multisystem biological risk, B(SE) = .93(.3), *p* = .002 after adjustment for sociodemographic factors, and remained significant after adjustment for health status (B(SE) = .79(.3), *p* = .009). Fig. 2 displays the adjusted means within sleep quality group by sleep duration categories. Secondary mixed linear effect analyses selecting only those cases reporting normal quality sleep demonstrated that long sleep duration compared to normal sleep duration was not associated with elevated multisystem risk among individuals reporting normal quality sleep (B(SE) = .17(.12), *p* = .14).

### Depression

As a secondary inquiry, we examined whether depressive symptoms might explain the elevated multisystem risk observed in short sleepers and among long sleepers with poor sleep quality. As Table 1 shows, 33% of short sleepers were in the high depressive symptoms group. However, further adjustment by depressive symptoms status did not alter the findings showing elevated multisystem risk in short sleepers (B(SE) = .42(.15), *p* = .005). Similarly, the percentage of subjects with high depressive symptoms in the long sleepers varied by sleep quality status, with 37.6% of poor sleep quality subjects reporting high depressive symptoms, while 8.6% of normal quality sleepers fell into the high depressive symptoms category (χ^2^ = 9.1, *p* < .005). Difference between poor sleep quality and normal sleep quality in multisystem biological risk among long sleepers remained significant after adjustment by depressive symptoms (B(SE) = .71(.31), *p* = .02).

Analyses examining associations of sleep duration and quality with each individual system risk score included in the multisystem biological risk index are available in the supplemental material, S2 Table.

## Discussion

The present set of analyses report significantly elevated multisystem biological risk scores in short sleepers, those reporting sleeping less than 5 hours a night, all of which report poor sleep quality. These finding are consistent with reports of elevated mortality risk is short sleepers and point to a possible mechanism through which inadequate sleep influences disease risk. The findings linking short sleep with elevated multisystem biological risk was retained after further adjustment by health status and depression suggesting that these factors do not account for the differences observed.

Our findings showing that inadequate sleep is associated with increased multisystem biological risk strengthens the existing research documenting similar associations of short sleep duration with a greater likelihood of exhibiting elevated biological risk across several individual regulatory systems (e.g., metabolic syndrome, hypertension, glucose regulation, immune, endocrine, autonomic). Likewise, the results are consistent with previous findings linking short sleep and sleep disturbances with allostatic load using measures that capture metabolic, cardiovascular, and immune systems [62,63]. However, the current study is the first to date to examine the relationship of sleep with multisystem biological risk, including sympathovagal and neuroendocrine parameters, and is comprised of 22 biomarkers specifically designed to capture allostatic load. These findings have significant clinical value given that over half of these biomarkers are commonly used individually to designate a unique disease risk (i.e., Hemoglobin A1c and diabetes) and are used together to predict morbidity and mortality outcomes [17,59–61].

Multisystem biological risk scores of long sleepers were also elevated compared to normal sleepers, but this difference was only in the long sleepers with poor sleep quality, an effect that remained after adjustment for depression. It was hypothesized that one possibility for elevated multisystem biological risk (and increased risk of mortality) in long sleepers is that individuals who sleep for longer periods than normal often have poorer sleep quality. Our result suggest that poor sleep quality in long sleepers contributes to elevated multisystem biological risk. This finding supports the hypothesis that too much sleep is associated with elevated risk because it may in fact serve as a proxy indicator of sleep fragmentation where individuals do not actually obtain the amount of sleep needed, which in turn increases the drive to sleep longer [8]. Under this hypothesis, sleep fragmentation could contribute to increased demands of the regulatory systems to maintain allostasis [14]. Since we were unable to directly test sleep apnea in the present analyses, it remains possible that our sample of long sleepers with poor sleep quality may include individuals with sleep apnea, and future research should consider this possibility.

Of particular note to medical professionals, these findings highlight the importance of sleep duration and the role of sleep quality as potential risk factors contributing to shifts in biological risk indicators. Similar to physician queries of other behavioral risk factors like smoking, low physical activity, and excess alcohol consumption, queries about sleep amounts and quality may be relevant [75].

### Mechanisms

A remaining question is why short sleep and poor sleep quality in long sleepers might impact these regulatory systems and how this might influence risk for disease. Sleep serves an important evolutionary function; it give the body time to rest and repair damage [75,76]. In the case of short sleep and sleep fragmentation, inadequate and disturbed sleep places demands on multiple regulatory systems throughout the body by reducing the available time for restoration and increasing the demand on physiological resources necessary to maintain stability (i.e., allostasis) [14]. Over time the accumulation of demands without appropriate restoration causes wear and tear, evidenced by allostatic load [15,16]. Thus prolonged periods of inadequate sleep likely contribute to gradual shifts in regulatory set points such as resting blood pressure, cholesterol, blood sugar, and inflammatory activity, which are themselves known to be involved in the pathophysiology of disease [55–57,77–79].

### Limitations and Strengths

Our findings are cross-sectional and thus do not show causation. Future work should focus on applying longitudinal and experimental designs to assess causality. Similarly, treatment trials to improve sleep in short sleepers and long sleepers with poor sleep quality are needed to examine whether multisystem biological risk improves along with remission of sleep problems. As previously stated, using self-reported sleep is limited and more objective measures of sleep continuity, along with a careful assessment for sleep apnea, would greatly improve our understanding of the links between short and long sleep with multisystem biological risk. There are numerous strengths to the current study including a large heterogeneous sample of adults in the US increasing the generalizability of the findings, and a comprehensive 22 biomarker measure of multisystem biological risk with established predictive validity for morbidity and mortality outcomes. In addition, the MIDUS study includes a detailed assessment of health, including an extensive assessment of self-reported chronic conditions, which allows us to control for the possible confound of health when examining relationships of sleep with biological risk. Likewise, the inclusion of a well validated and reliable measure of depressive symptoms (the CESD) [72,73] adds to the strength of the current analyses revealing that further statistical adjustments for depression does not account for the differences in multisystem biological risk among short sleepers and long sleepers with poor sleep quality.

## Conclusions

In sum, we report in a large community based sample of adults living in the Unites States of America that similar to findings linking sleep duration with mortality risk, short sleepers and long sleepers with poor sleep quality had elevated biomarkers of risk indicative of multisystem dysregulation. This finding was independent of sociodemographics, chronic health conditions, self-evaluated health, and depression. The association of long sleepers with elevated multisystem biological risk was only present in those reporting poor sleep quality, suggesting that self-reported long sleep may be related to disease partially because it captures poor sleepers. Poor and inadequate sleep may contribute to dysregulation in physiological set points across numerous regulatory systems, represented as allostatic load. These findings suggest that acquiring adequate amounts of good quality sleep (at least greater than 5 hours) is associated with less multisystem biological risk, and may therefore also be a means to lower risk for morbidity and mortality. Individuals who report global poor sleep quality (>5 on PSQI), either with long sleep or short sleep, are also experiencing some insomnia symptoms. Meta-analytic evidence suggests that both insomnia symptoms and global sleep quality are improved with behavioral interventions (e.g., cognitive behavioral therapy for insomnia, CBT-I) [61], suggesting that such treatments may also be useful in improving sleep quality even among those who do not meet full criterion for insomnia disorder. Future research should examine the treatment efficacy of CBT-I and possibly other behavioral interventions for subsyndromal insomnia as indicated by high global PSQI scores with short or long sleep duration. In the case of long sleep, current interventions are underway to target sleep restriction in long sleepers as a means to improve health, which may act by resetting diurnal rhythms [80]. The present findings point to the importance of evaluating sleep amounts and quality during assessment of lifestyle factors associated with cardiovascular and other disease risk.

## Supporting Information

S1 Table Correlation coefficients between subscales of the multisystem biological risk index. (DOCX)

S2 Table Results of linear mixed model analyses examining associations of individual system scores with sleep parameters. (DOCX)
