# Supplementary material for: Sleep and Multisystem Biological Risk: A Population-Based Study
Source: PLoS One. 2015 Feb 25;10(2):e0118467. doi: 10.1371/journal.pone.0118467 (PMC4340787; doi:10.1371/journal.pone.0118467)
Supplement: S1 Table — (DOCX) [file pone.0118467.s001.docx]

**S1 Table.** **Correlation coefficients between subscales of the multisystem biological risk index.**

|  | 1 | 2 | 3 | 4 | 5 | 6 |
| --- | --- | --- | --- | --- | --- | --- |
| 1. Sympathetic subscale |  |  |  |  |  |  |
| 2. Parasympathetic subscale | .134^**^ |  |  |  |  |  |
| 3. HPA axis subscale | .146^**^ | .071^*^ |  |  |  |  |
| 4. Inflammation subscale | .099^**^ | .132^**^ | -.030 |  |  |  |
| 5. Cardiovascular subscale | .215^**^ | .233^**^ | .147^**^ | .247^**^ |  |  |
| 6. Metabolic-glucose subscale | .021 | .154^**^ | -.030 | .309^**^ | .286^**^ |  |
| 7. Metabolic-lipids subscale | -.067^*^ | .130^**^ | -.114^**^ | .211^**^ | .207^**^ | .415^**^ |
| **Significant at the 0.01 level (2-tailed) | | | | | | |
| *Significant at the 0.05 level (2-tailed) | | | | | | |
