# Supplementary material for: Sleep and Multisystem Biological Risk: A Population-Based Study
Source: PLoS One. 2015 Feb 25;10(2):e0118467. doi: 10.1371/journal.pone.0118467 (PMC4340787; doi:10.1371/journal.pone.0118467)
Supplement: S2 Table — (DOCX) [file pone.0118467.s002.docx]

**S2 Table. Results of linear mixed model analyses examining associations of individual system**

**scores with sleep parameters.**

|  | Short vs. Normal | | Long vs. Normal | | Poor vs. Good Sleep Quality | |
| --- | --- | --- | --- | --- | --- | --- |
|  | B | SE | B | SE | B | SE |
| 1. Sympathetic subscale | -0.07 | 0.05 | -0.02 | 0.04 | 0.01 | 0.02 |
| 2. Parasympathetic subscale | -0.05 | 0.06 | -0.13*** | 0.04 | -0.02 | 0.02 |
| 3. HPA axis subscale | -0.003 | 0.04 | 0.06 | 0.03 | -0.01 | 0.02 |
| 4. Inflammation subscale | -0.09* | 0.04 | -0.06* | 0.03 | -0.03 | 0.05 |
| 5. Cardiovascular subscale | -0.12** | 0.05 | -0.03 | 0.03 | -0.01 | 0.02 |
| 6. Metabolic-glucose subscale | -0.15** | 0.05 | -.10** | 0.04 | -.08*** | 0.02 |
| 7. Metabolic-lipids subscale | 0.02 | 0.03 | -.06** | 0.02 | -0.02 | 0.01 |

Note: Single system analyses typically produce smaller estimates than the multisystem index that examines combined

system effects. Interpretation of effect sizes within individual systems should not be used as a justification to target

only those systems and disregard the multisystem approach. Models adjust for age, gender, BMI, and socioeconomic status.

****p*<.001; ***p*<.01; **p*<.05
